# Supplementary figures and images for: The green, gold grass of home: Introducing open access in universities in Norway
Source: PLoS One. 2022 Aug 17;17(8):e0273091. doi: 10.1371/journal.pone.0273091 (PMC9385055; doi:10.1371/journal.pone.0273091)

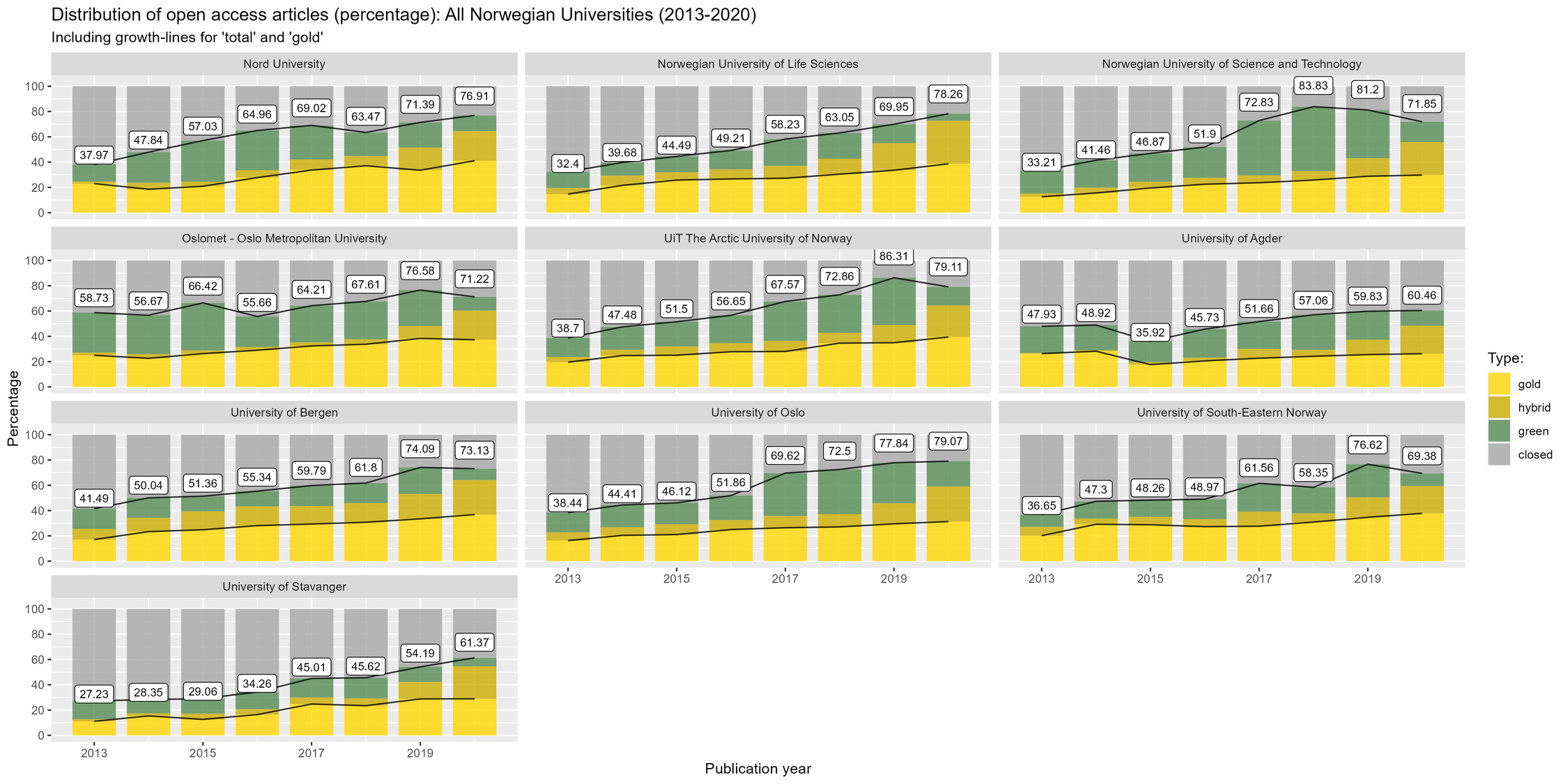

Supplement: S1 Fig — Overview of gold open access, hybrid open access and green open access in the period 2013–2020 given in percentage. Upper line indicate growth in total levels of open access, bottom line indicate growth in gold open access. (TIF) [file pone.0273091.s001.tif]
