# Supplementary material for: The green, gold grass of home: Introducing open access in universities in Norway
Source: PLoS One. 2022 Aug 17;17(8):e0273091. doi: 10.1371/journal.pone.0273091 (PMC9385055; doi:10.1371/journal.pone.0273091)
Supplement: Attachment — Submitted filename: Response to Reviewers.docx [file pone.0273091.s002.docx]

Dear editor and reviewers

First, we would like to express our gratitude for the comments and viewpoints from both the editor and the reviewers. In general, we agree that dealing with the proposed changes indeed will raise the quality of the manuscript. In this document we comment on each of the editor’s and reviewers’ points. The list is slightly condensed since some of the issues are mentioned more than once but will follow the order given in the response letter from the editor.

We hope that by addressing these issues, the manuscript will meet the standards of PLOS ONE and be accepted for publication.

**Editor’s comments:**

- the structure of the paper should be re-thought at certain points so that it possesses more consistency.

**Response**: We have rewritten the review and background section according to the remarks from the reviewers, particularly the point made about consistency. The logic is now to start with the open access literature, moving on to the literature on how universities institutionalise new practices (like open access and especially tied to the third mission), and finally tying this part together by reviewing the (small) relevant literature on how open access has emerged in Norway. We have furthermore added and removed sentences in other parts of the manuscript to make the structure of the arguments clearer. See also comments below.

- As Reviewer 2 points out, you should be more concrete about why you worked with these ten universities of your choice and place them in the larger context of Norwegian higher education.

**Response**: All Norwegian universities were included in the study, and we acknowledge that this should be made clearer (since it may also be seen as a strength of the study). We have specified that our ten universities represent the whole population of universities in Norway several places, and we have added a few sentences at the end to discuss what we might have found if other research institutions (colleges, institutes) had been included. (Also commented below along with the remarks made by reviewer #2.)

- Both reviewers commented on the literature review, probably a separate section on this would help.

**Response**: This point is connected to the first point in the bullet-list and is also commented below. We have added some more references to earlier investigations and restructured the literature review. Since we have found no earlier studies about the institutionalisation of open access in universities apart from the two references already in the text, our aim has been to strengthen the review of open access without going into detail into general studies of it, which seem too much on the side of our aim and research question.

1. Please ensure that your manuscript meets PLOS ONE's style requirements, including those for file naming. The PLOS ONE style templates can be found at 
<https://journals.plos.org/plosone/s/file?id=wjVg/PLOSOne_formatting_sample_main_body.pdf> and 
<https://journals.plos.org/plosone/s/file?id=ba62/PLOSOne_formatting_sample_title_authors_affiliations.pdf>.
**Response**: The style guide has again been consulted and we believe that the manuscript adheres to the PLOS One requirements.

2. Thank you for stating the following in the Competing Interests section: 
[I have read the journal's policy and the authors of this manuscript have the following competing interests: Lars Wenaas is a PhD-candidate at the TIK-center at the University of Oslo while holding a position at 'Sikt', a governmental body reporting to the ministry of education and research in Norway. The position is a part of the department with responsibility of coordinating open access affairs in Norway.]

Please confirm that this does not alter your adherence to all PLOS ONE policies on sharing data and materials, by including the following statement: "This does not alter our adherence to  PLOS ONE policies on sharing data and materials.” (as detailed online in our guide for authors <http://journals.plos.org/plosone/s/competing-interests>). If there are restrictions on sharing of data and/or materials, please state these. Please note that we cannot proceed with consideration of your article until this information has been declared.

Please include your updated Competing Interests statement in your cover letter; we will change the online submission form on your behalf.
**Response**: The competing interest statement has been updated in the cover letter with PLOS ONE policy on sharing of data.

3. We note that you have stated that you will provide repository information for your data at acceptance. Should your manuscript be accepted for publication, we will hold it until you provide the relevant accession numbers or DOIs necessary to access your data.
**Response**: Since the original submission, the data set has been published. The referenced DOI at line 371 is now a valid URL. We have also included the dataset as a reference in the reference section.

4. We note you have included a table to which you do not refer in the text of your manuscript. Please ensure that you refer to Table 1 in your text; if accepted, production will need this reference to link the reader to the Table.
**Response**: The text now refers to Table 1.

5. Please review your reference list to ensure that it is complete and correct. If you have cited papers that have been retracted, please include the rationale for doing so in the manuscript text, or remove these references and replace them with relevant current references. Any changes to the reference list should be mentioned in the rebuttal letter that accompanies your revised manuscript. If you need to cite a retracted article, indicate the article’s retracted status in the References list and also include a citation and full reference for the retraction notice.
**Response**: The reference list has been reviewed and several new references has been added as part of the changes in the review-section.

**Reviewer #1:** 

1. The topics of the three sections of the literature review are separated strongly. So, there is a considerable thematic gap between the consecutive parts. The content of the sections should be linked consistently. It is also worth considering whether the sequence of the three sections of the literature review is appropriate.
**Response**: We agree with the view of the reviewer and have reorganised and reordered the review section to make it more coherent.

2. In the second section of the literature review, the authors should also address how open access and the quality (or rankings) of academic journals are related to each other and whether there are any differences among disciplines regarding open access publishing.
**Response**: We have added remarks (starting at line 137) on the relationship between journals rankings and open access together with disciplinary differences in the review-part of the manuscript.

3. The authors found little information about the advantages of open access for education in the studied documents. However, more details should be provided about how open access can support education. Some statements and remarks would be useful and may be incentive.
**Response**: We have expanded on this topic in the review-part of the manuscript, starting at line 149.

4. Why open access publishing can be advantageous for researchers should also be pointed out.
**Response**: We have added more comments on this topic in the literature review part of the manuscript, starting at line 133.

5. The manuscript does not explain the issues of open access publishing funds. However, these are also relevant for universities and researchers (as the authors of papers). How universities cover the article processing charges (APC) and distribute the (dedicated) open access publishing funds among disciplines, departments, research projects, and researchers are crucial questions. It is also relevant how researchers can require financial resources to cover APC. These issues are also related to institutionalization processes and should be discussed in the manuscript.
**Response**: This is potentially a large issue that the authors have discussed thoroughly during the study. We decided then to not dedicate much space to funds, as funds primarily belong to the infrastructure and practises of open access, rather than how the universities perceive and integrate open access in their portfolio of tasks. In addition, dedicated funds are linked to gold open access only. We do however agree with the reviewer that it can be seen as relevant to the institutionalisation processes, and we have therefore incorporated some text about institutional funds and how we view their relevance in our analysis of the empirical data. (Starting at line 329).

Minor issues
1. The main title of the manuscript should be worded more professionally.
**Response**: We have thought carefully about this. The current first part of the title contains both a reference to two the two main types of open access and an allusion to a famous pop song (Tom Jones, 1967) that eludes the “heartland” concept which is important in our analysis. If the editor asks us to, we are happy to provide a more conservative title such as “Institutionalising open access in Norwegian universities”.

2. The time frame of the study should be indicated in the abstract.
**Response**: The time frame is now included in the abstract.

3. Single and double quotations marks are applied alternately in the text. A coherent format is required.
**Response**: Quotations marks are now coherent throughout the manuscript.

4. The page number is missing in some word by word citations, e.g. on page 5, in lines 191-193. It is required in each quotation.
**Response**: We have added page numbers to all relevant quotations beyond a couple of words/concepts and where the referenced object is a webpage.

5. The authors should give some remarks in the conclusion that could be useful for other countries and universities.
**Response**: We have added a few sentences that reflect upon the usefulness for other countries, starting at line 801.

6. Notes should be provided in Figure 1 to understand the markings clearly.
**Response**: The explanation of markings was separated from the figure but is given in the figure label.

7. In Figure 2, all the signs have to be given in English.
**Response**: The university names have been translated to English.

**Reviewer #2:**

1. [..] it should be emphasized better, why not all Norwegian universities are included.
**Response**: See earlier comment. All Norwegian universities were actually included in the study, and we have made this clearer and more explicit.

2. Also, the literature review part should be established. Now we can see the theoretical framework but not an overview, what other authors wrote about this topic. It would be an essential issue, as the topic itself is very young. So, in this way, a critical-analytical, comprehensive literature review would be one of the strengths of the paper and could generate a more wide publicity.
**Response**: We have rewritten the literature review part to embed the theoretical framework better and also to start with a slightly larger review of the open access literature. However, we have not found any relevant studies directly dealing with the institutionalisation of open access, but we have nevertheless attempted to make the review more coherent in how it covers main themes from the open access literature. It is possible to add a more general text about institutionalisation, however we believe the paper as it now stands will be more to the point for the readers, with clear choices made on relevant studies rather than a more generic text.
